# Supplementary material for: Equivalent current dipole sources of neurofeedback training-induced alpha activity through temporal/spectral analytic techniques
Source: PLoS One. 2022 Feb 25;17(2):e0264415. doi: 10.1371/journal.pone.0264415 (PMC8880644; doi:10.1371/journal.pone.0264415)
Supplement: Attachment — Submitted filename: response to reviewers PONE-D-20-20804.docx [file pone.0264415.s001.docx]

Dear Editor-in-Chief:

This manuscript is a resubmission of the manuscript PONE-D-19-25083. Previous Academic Editor (Thalia Fernandez, Ph.D.) suggested that the previous manuscript must be submitted in an intelligible manner and be written in standard English because it has some grammatical errors. Therefore, we send it for editing with a native English speaker by American Journal Experts (AJE) and resubmission.
