# Supplementary material for: Equivalent current dipole sources of neurofeedback training-induced alpha activity through temporal/spectral analytic techniques
Source: PLoS One. 2022 Feb 25;17(2):e0264415. doi: 10.1371/journal.pone.0264415 (PMC8880644; doi:10.1371/journal.pone.0264415)
Supplement: Attachment — Submitted filename: Review of PONE-D-20-20804 by A. Zani_Sep 20, 2020.docx [file pone.0264415.s002.docx]

The present study aimed to identify sources of training-induced alpha activity through four different temporal/spectral analytic techniques, relating the effects of the training to ”memory function”.

As I understood, the authors have already reviewed their paper. In my opinion, the authors did a remarkable work, I also guess thanks to the valuable reviewers' feedback.

The paper now is very well structured, with a properly deep introduction and large and detailed discussions.

Undoubtedly, I believe some weaknesses still affect the work, most of all the generic reference of alpha power in their study to memory functions without directly testing this reference by means of a specific memory task used in memory studies carried out in the context of cognitive neuroscience literature on this matter.

Indeed, I was most surprised of finding this reference in the light of the kind of procedure to whom their experimental participants were submitted, namely a neurofeedback training (NFT) to self-regulate their own brain activity. Indeed, rather than a mnemonic task, this can be referred to as a learning to self-regulate their own brain alpha activity by means of a progressive increase of individual cognitive control on brain neural networks subserving alertness and focusing of attention functions.

However, I believe that this may not be hard to solve now. Indeed, I think that the hard work made so far by the authors would deserve one more chance to achieve an acceptable level and be published.

Indeed, the EEG literature is plenty of studies directly relating alpha power to the functions of alerting, orienting, and cognitive control characterizing the different neural networks of the visual selective attention system. Below, for instance, I reported a short list of recent and less recent studies in the literature on alpha power and visual attention to be used as a starting point for a critical review of their findings and of those papers referred to by these studies:

Zani, A.; Tumminelli, C.; Proverbio, A.M. Electroencephalogram (EEG) Alpha Power as a Marker of Visuospatial Attention Orienting and Suppression in Normoxia and Hypoxia. An Exploratory Study. *Brain Sci.* **2020**, *10*, 140.

Misselhorn, J.; Friese, U.; Engel, A.K. Frontal and parietal alpha oscillations reflect attentional modulation of cross-modal matching. *Sci. Rep.* **2019**, *22*, 5030.

Rihs, T.A.; Michel, C.M.; Thut, G. Mechanisms of selective inhibition in visual spatial attention are indexed by alpha-band EEG synchronization. *Eur. J. Neurosci.* **2017**, *25*, 603–610.

Poch, C.; Carretie, L.; Campo, P. A dual mechanism underlying alpha lateralization in attentional orienting to mental representation. *Biol. Psychol.* **2017**, *28*, 63–70.

Foxe, J.J.; Snyder, A.C. The role of alpha-band brain oscillations as a sensory suppression mechanism during selective attention. *Frnt. Hum. Neurosci.* **2011**, *2*, 154.

Kelly, S.P.; Lalor, E.C.; Reilly, R.B.; Foxe, J.J. Increases in Alpha Oscillatory Power Reflect a Suppression during Sustained Visuospatial Attention Active Retinotopic Mechanism for Distracter. *J. Neurophysiol.* **2006**, *95*, 3844–3851.

As I already wrote, I believe that if the authors will be able to eliminate any reference to generic and abstract “memory functions” both in the Abstract and in any other paper sections, mostly the Introduction and Discussion sections, and to provide a short critical discussion of their NFT alpha findings in the light of the attentional findings advanced in the indicated studies, the paper will be more than worth to be published.

As a more specific point, please provide a very short explanation of the NFT procedure already in the Abstract.
