# Supplementary material for: Equivalent current dipole sources of neurofeedback training-induced alpha activity through temporal/spectral analytic techniques
Source: PLoS One. 2022 Feb 25;17(2):e0264415. doi: 10.1371/journal.pone.0264415 (PMC8880644; doi:10.1371/journal.pone.0264415)
Supplement: Attachment — Submitted filename: Response to Reviewers.docx [file pone.0264415.s003.docx]

PONE-D-20-20804

Equivalent current dipole sources of neurofeedback training-induced alpha activity through temporal/spectral analytic techniques.

PLOS ONE

Dear Dr. Shaw,

Thank you for submitting your manuscript to PLOS ONE. After careful consideration, we feel that it has merit but does not fully meet PLOS ONE’s publication criteria as it currently stands. Therefore, we invite you to submit a revised version of the manuscript that addresses the points raised during the review process.

Please submit your revised manuscript by Nov 06 2020 11:59PM. If you will need more time than this to complete your revisions, please reply to this message or contact the journal office at plosone@plos.org. When you're ready to submit your revision, log on to https://www.editorialmanager.com/pone/ and select the 'Submissions Needing Revision' folder to locate your manuscript file.

Please include the following items when submitting your revised manuscript:

- A rebuttal letter that responds to each point raised by the academic editor and reviewer(s). You should upload this letter as a separate file labeled 'Response to Reviewers'.
- A marked-up copy of your manuscript that highlights changes made to the original version. You should upload this as a separate file labeled 'Revised Manuscript with Track Changes'.
- An unmarked version of your revised paper without tracked changes. You should upload this as a separate file labeled 'Manuscript'.

If you would like to make changes to your financial disclosure, please include your updated statement in your cover letter. Guidelines for resubmitting your figure files are available below the reviewer comments at the end of this letter.

If applicable, we recommend that you deposit your laboratory protocols in protocols.io to enhance the reproducibility of your results. Protocols.io assigns your protocol its own identifier (DOI) so that it can be cited independently in the future. For instructions see: <http://journals.plos.org/plosone/s/submission-guidelines#loc-laboratory-protocols>

We look forward to receiving your revised manuscript.

Kind regards,

Alice Mado Proverbio

Academic Editor

PLOS ONE

Journal Requirements:

When submitting your revision, we need you to address these additional requirements.

1. Please ensure that your manuscript meets PLOS ONE's style requirements, including those for file naming. The PLOS ONE style templates can be found at

https://journals.plos.org/plosone/s/file?id=wjVg/PLOSOne_formatting_sample_main_body.pdf and

https://journals.plos.org/plosone/s/file?id=ba62/PLOSOne_formatting_sample_title_authors_affiliations.pdf

2. In your Methods section, please provide additional information about the participant recruitment method and the demographic details of your participants. Please ensure you have provided sufficient details to replicate the analyses such as: a) the recruitment date range (month and year), b) a description of any inclusion/exclusion criteria that were applied to participant recruitment, c) a table of relevant demographic details, d) a statement as to whether your sample can be considered representative of a larger population, e) a description of how participants were recruited, and f) descriptions of where participants were recruited and where the research took place.

**Response:** Thank you for the editor’s suggestion. In this version, we have provided demographic details (Table 1) and other information mentioned above in the “Materials and methods” section.

(lines 89-95) “In this study, the participants recruited via social media, e.g., Facebook and Instagram, included 63 healthy students studying at National Cheng Kung University from May 2016 to March 2018 using convenience sampling, and they were randomly assigned to two age- and sex-matched groups. The inclusion criteria were Taiwanese nationality, 20-30 years old, right-handed and not having participated in an NFT study in the past. The exclusion criteria included a history of mental or neurological disorders and potential pregnancy.”

(line 122) “All of the experiments were performed in our laboratory.”

3. Thank you for your ethics statement:

'The experimental procedure was reviewed and approved by a local research ethics committee. Informed consent was provided and signed by all participants before the experiment.'

Please amend your current ethics statement to include the full name of the ethics committee/institutional review board(s) that approved your specific study.

**Response:** Thank you for the editor’s suggestion. In this version, the full name of the ethics committee/institutional review board is given as the Institutional Review Board of the National Cheng Kung University Hospital (NCKUH-IRB). (lines 101-103)

Once you have amended this/these statement(s) in the Methods section of the manuscript, please add the same text to the “Ethics Statement” field of the submission form (via “Edit Submission”).

For additional information about PLOS ONE ethical requirements for human subjects research, please refer to http://journals.plos.org/plosone/s/submission-guidelines#loc-human-subjects-research.

4. Thank you for stating the following in the Acknowledgments Section of your manuscript:

"The authors thank the following institutions for their research support: Ministry of Science and Technology, Taiwan (108-2410-H-006-112-MY3 and 108-2634-F-006-012), and the Mind Research and Imaging Center, National Cheng Kung University, Taiwan."

We note that you have provided funding information that is not currently declared in your Funding Statement. However, funding information should not appear in the Acknowledgments section or other areas of your manuscript. We will only publish funding information present in the Funding Statement section of the online submission form.

**Response:** Thank you for the editor’s suggestion. In this version, we have removed funding information from the “Acknowledgments” section.

Please remove any funding-related text from the manuscript and let us know how you would like to update your Funding Statement. Currently, your Funding Statement reads as follows:

"The author(s) received no specific funding for this work."

Please include your amended statements within your cover letter; we will change the online submission form on your behalf.

[Note: HTML markup is below. Please do not edit.]

Reviewers' comments:

Reviewer's Responses to Questions

**Comments to the Author**

1. Is the manuscript technically sound, and do the data support the conclusions?

The manuscript must describe a technically sound piece of scientific research with data that supports the conclusions. Experiments must have been conducted rigorously, with appropriate controls, replication, and sample sizes. The conclusions must be drawn appropriately based on the data presented.

Reviewer #1: Partly

Reviewer #2: Partly

Reviewer #3: No

Reviewer #4: Partly

2. Has the statistical analysis been performed appropriately and rigorously?

Reviewer #1: Yes

Reviewer #2: No

Reviewer #3: I Don't Know

Reviewer #4: Yes

3. Have the authors made all data underlying the findings in their manuscript fully available?

The [PLOS Data policy](http://www.plosone.org/static/policies.action#sharing) requires authors to make all data underlying the findings described in their manuscript fully available without restriction, with rare exception (please refer to the Data Availability Statement in the manuscript PDF file). The data should be provided as part of the manuscript or its supporting information, or deposited to a public repository. For example, in addition to summary statistics, the data points behind means, medians and variance measures should be available. If there are restrictions on publicly sharing data—e.g. participant privacy or use of data from a third party—those must be specified.

Reviewer #1: No

Reviewer #2: Yes

Reviewer #3: Yes

Reviewer #4: Yes

4. Is the manuscript presented in an intelligible fashion and written in standard English?

PLOS ONE does not copyedit accepted manuscripts, so the language in submitted articles must be clear, correct, and unambiguous. Any typographical or grammatical errors should be corrected at revision, so please note any specific errors here.

Reviewer #1: Yes

Reviewer #2: Yes

Reviewer #3: Yes

Reviewer #4: Yes

**5. Review Comments to the Author**

Please use the space provided to explain your answers to the questions above. You may also include additional comments for the author, including concerns about dual publication, research ethics, or publication ethics. (Please upload your review as an attachment if it exceeds 20,000 characters)

Reviewer #1: The manuscript titled “Equivalent current dipole sources of neurofeedback training-induced alpha activity through temporal/spectral analytic techniques” touches upon evergreen subject of EEG neurofeedback. It comes with reasonable number of participants (63) and an interesting attempt to localize sources of observed EEG activity.

Although interesting some major questions need to be answered before final conclusions can be made:

1. The training protocol was based on the alpha band which is highly susceptible to various manipulation (Williams, 1977; Quandt et al. 2012) and the training protocol included instructions for participants. How many trainers did provide those instructions and how authors assured mitigation of trainer effect?

**Response:** Thank you for the reviewer’s suggestion. In this study, only one trainer provided instructions and strategies and trained all of the participants. Before the experiment, the trainer was trained to be familiar with the training protocol, ensuring that the trainer could provide instructions and strategies to each participant consistently during training.

2. Were the participants personality profiles investigated (using psychological tests such as EPQ-R, NEO-EFI,…) ?

**Response:** Thank you for the reviewer’s suggestion. In this study, we did not investigate the participants’ personality profiles. This issue is an interesting one for future NFT research.

Authors’ results are based only on the “responders” subgroup of the “alpha” - experimental group：

1. How many exactly “responders” were finally identified?

**Response:** Thank you for the reviewer’s suggestion. In this study, twenty-nine participants (83%) were identified as “responders.” (line 261).

2. Are there any specific features allowing to distinguish responders vs non-non-responders other than increase in alpha amplitude?

**Response:** Thank you for the reviewer’s suggestion. Yes, in our previous study [1], we used total alpha duration (all successful 1-s events within a session were accumulated) to distinguish responders.

3. Is there any relations between responders, non-responders and instructions, trainers or personality traits?

**Response:** Thank you for the reviewer’s suggestion. In this study, only one trainer trained all of the participants with consistent instructions. We did not investigate the participants’ personality profiles. There were no relations of responders and non-responders with instructions, trainers or personality traits. This issue is also another interesting one for future NFT research.

4. What would be result for comparison all “alpha” (experimental) group vs “ctrl” (control) group?

**Response:** Thank you for the reviewer’s suggestion. No significant difference was observed in mean alpha amplitude of the Ctrl group throughout the 12 sessions. Only the all-“alpha” (experimental) group showed a progressively increasing change in alpha amplitude throughout the training sessions. The result (see below) was similar to that in Fig. 1. We focused on well-trained “responders” in the present study. Therefore, we only presented the data of “responders” in the Alpha group.

Mean relative alpha amplitude changes throughout the 12 sessions in the all-“Alpha” (experimental) group and “Ctrl” (control) group. The gray line is a linear regression line and indicates a progressive, linear increase in the Alpha group. * *p* < 0.05 vs. the 1st session. ^#^ *p* < 0.05 vs. the Ctrl group.

Authors used bipolar electrode montage very rarely used in this kind of investigations which makes it difficult to compare to similar studies:

1. Why Authors decided to use bipolar montage?

**Response:** Thank you for the reviewer’s suggestion. In the present study, a bipolar electrode montage was only used in the neurofeedback training and not whole-head EEG. Bipolar recording was beneficial to reduce the possible artifacts of motion or eye blinks. The purpose (either bipolar montage or other montage of other NFT studies) is to calculate changes in alpha activity during training. It would be interesting to investigate differences between montages for future research.

2. What would be the results recalculated to the average reference?

**Response:** Thank you for the reviewer’s suggestion. The EEGs of six electrodes were converted into three bipolar EEGs in the amplifier circuit board. Three bipolar EEGs were digitized by an analog-to-digital converter to computer and were acquired by LabVIEW software. Therefore, there are no original EEGs of six electrodes that can be recalculated to the average reference.

3. What was the results of whole head analyses for “non-responders”?

**Response:** In this study, only well-trained “responders” in the Alpha group participated in whole-head EEG recording. “Non-responders” did not meet the criteria of “responders” and did not participant in whole-head EEG recordings. Therefore, there are no results of whole head analyses for “non-responders.”

Source analyses

1. Sources were calculated using Curry software which the procedure is not publicly available, could you provide detailed methodology, please?

**Response:** There have been many studies using Curry software (transition from Scan software) to conduct dipole source localization. Generally, of course, a qualified signal is needed first; the procedure includes deciding on the number of dipoles used for dipole source localization (depending on assumptions according to neurophysiological and anatomical knowledge from previous studies) and choosing a head model (e.g., spherical or BEM) and dipole model (e.g., regional or LORETA). However, no recent study has provided information about the sources of training-induced alpha activity. Therefore, we used ICA to define the numbers of dipoles. The number of dipoles used for dipole source localization depended on the number of valid components (SNR > 1) [20]. Dipole source localization was conducted for each “responder.” Subsequently, we projected the dipoles onto the brain.

2. From my understanding sources were calculated using ICA. Since ICA will return different clusters for each participant how the results were averaged: calculated individually, finding common clusters and then averaged or they were all subjects were analyzed together?

**Response:** Thank you for the reviewer’s suggestion. The ICA and dipole source localization was performed for each “responder.” In this study, the purpose of using ICA was simply to define the numbers of dipoles used in dipole source localization. For each “responder,” the averaged alpha epoch was applied in ICA before dipole source localization. Several ICA components (depending on the number of channels) were estimated, and we calculated their SNR. The number of dipoles used for dipole source localization depended on the number of valid components (SNR > 1). When the number of dipoles was decided, it was used for dipole source localization of the averaged alpha epoch. In this revised version, we have reorganized the description in the dipole source localization part of the “Materials and methods” section.

(lines 215-222) “*In the present study, independent component analysis (ICA) was used to define the numbers of dipoles, and dipole source localization was performed for each “responder.” ICA source reconstruction was conducted to compute a fixed dipole source for each independent component [19]. The averaged alpha epoch was applied in ICA before dipole source localization. ICA components were estimated, and we calculated their signal-to-noise ratio (SNR). The number of dipoles used for dipole source localization depended on the number of valid components (SNR > 1) [20].”*

3. Without individual electrode positions and/or individual T1s localization results will not provide accurate results allowing for anatomical considerations

**Response:** Thank you for the reviewer’s suggestion. We had individual electrode positions. In this study, all of the electrode positions were measured using a 3D digitizer (Fastrak, Polhemus, USA) for dipole source analyses. The description has been added to the whole-head EEG and processing part of the “Materials and methods” section (lines 169-170). In contrast, we did not acquire individual 3D MRI data but used standardized BEM models to conduct source localization. Indeed, individual MRI data can improve the source localization accuracy. However, source localization using EEG is used to estimate approximate brain regions. Fuchs et al. (2004) (https://doi.org/10.1016/S1388*-*2457(02)00030-5) reported that no significant differences in source localization were observed and showed the similarity in source localization between individual and standardized BEM models. Moreover, the standardized BEM models showed significantly better localization accuracy than the individual BEM model. The evidence revealed that standardized BEM models remain sufficient for source localization. Certainly, there are several factors affecting the accuracy of source localization, e.g., different brain activities, head models and source localization methods. For training-induced alpha activity, these issues would be interesting for future research.

Other methodological questions:

1. Authors investigated also the delta, theta and beta (13-30 Hz) bands what was the results how they compare to alpha band results (especially the theta band which could possibly drive the alpha one)?

**Response:** Regarding the reviewer’s concern, we calculated the amplitude difference between 12^th^ and 1^st^ sessions for each band. The paired t-test revealed that the amplitude difference of the alpha band was significantly greater than that of delta (*p* = 0.005), theta (*p* = 0.007) and beta (*p* < 0.001). No significant amplitude differences were observed between delta and theta (*p* = 0.176), delta and beta (*p* = 0.573), and theta and beta (*p* = 0.291). The results were the same as the results when we compared the amplitudes of these bands between the 1st and 12th sessions (lines 282-284). These data indicated that no amplitude of bands was increased, except for the alpha band after NFT.

2. What was exactly calculation of ERSPA?

**Response:** The calculation of ERSPA used the ERSP technology to measure amplitude spectra at each time point for each categorized alpha epoch, and then amplitude spectra were averaged over time. An inverse FFT was applied to averaged ERSP to generate a time series of averaged alpha epoch. The description has been added to the whole-head EEG and processing part of the “Materials and methods” section.

(lines 199-202) *“**The ERSP technology measured amplitude spectra at each time point. The amplitude spectra of each categorized alpha epoch were averaged over time. An inverse FFT was applied to the averaged ERSP to generate a time series of averaged alpha epoch.”*

**Reviewer #2**: The authors aimed to explore the neurophysiological sources of alpha increases induced by neurofeedback training. Therefore, they calculated the power increases during 12 sessions of alpha neurofeedback training by using four different analysis techniques, namely the maximum peak average, the positive average, the negative average, and the event-related spectral perturbation average. All analysis methods showed a high agreement on localized dipole sources in the precuneus, posterior cingulate cortex, and middle temporal gyrus. The authors conclude that all measures are suitable for investigating dipole sources during neurofeedback training.

The research question itself is an interesting one and worth to be investigated. However, it is unclear how the authors derived the research question and why the described methods were chosen in order to answer it. The method section lacks important descriptions and it is not comprehensible why and on the basis of which criteria the authors ended up with a source localization analysis with 14 participants despite 63 that were tested. It is questionable why four analysis techniques were compared, considering that the research question focused on exploration of the source regions of alpha activity, and it is not reported whether source localization would have been significant using solely one of these methods. Furthermore, source localization results differ between text and table. All together, it remains unclear what the results contribute to the understanding and future conduction of alpha neurofeedback training and its effectiveness to enhance cognitive performance. Therefore, the manuscript needs to be substantially revised before to be considered for publication.

Major points:

1. It is not clear on what basis the research question was derived and why it is important to explore it. The introduction is rather confusing and it is not convincingly reported why the authors investigated dipole sources in EEG activity that was measured after neurofeedback training and not during training. Transfer of neurofeedback training to resting-state activity in previous studies has been mixed and EEG activity after training could simply display fatigue and not the training effect itself (as there was no feedback during the alpha blocks of the whole-head EEG). Moreover, it is not apparent why and how the authors chose the four analysis techniques and compared them. I was wondering why the authors did not use one of the more conventional source localization methods like beamforming or LORETA. The abstract even mentions that alpha neurofeedback training is important for memory function but this focus (or generally transfer of neurofeedback training) is not explored at all (only mentioned in the discussion, please see point 6).

**Response:**

Regarding the reviewer’s concerns, we have reorganized the introduction to clarify the research question (lines 36-79). The purpose of this study was to investigate the generation of training-induced alpha activity. During training, participants still learned how to successfully enhance their alpha activity. As in Fig. 1, alpha amplitude still did not significantly increase in the initial training session, showing that the participants were still unstable to enhance alpha activity. After 12 training sessions, we found that most of the participants could successfully enhance and increase alpha activity. Furthermore, we chose “responders” to conduct whole-head EEG recording and extract successful alpha trials (> 1.5 averaged alpha amplitude of EEGs of fixation blocks) for dipole source localization. Indeed, there was no feedback during whole-head EEG recording. However, our “responders” reported that they had learned a strategy to enhance alpha activity after NFT. They had enhanced alpha activity in alpha blocks of whole-head EEG, resulting in approximately 250-500 successful alpha trials (each “responder”), which were used for analysis. Indeed, the temporal impact of NFT on brain activity is an interesting issue for future NFT research.

There are two types of localization methods. One is a distributed method (such as LORETA or beamformer), and the other one is a dipolar method (such as regional dipole). The disadvantages of LORETA are low spatial resolution and blurred localized images of a point source with dispersion in the image (Asadzadeh et al., 2020) (https://doi.org/10.1016/j.jneumeth.2020.108740). The distributed method is an area-wise localization. In contrast, the dipolar method is a point-wise localization. When the dipole number is unknown, the dipolar method is suitable for source localization (Sonoda et al., 2018) (https://doi.org/10.1016/j.neunet.2018.08.008). There are still no assumptions helping us to decide the dipole number used for dipole fitting in training-induced alpha activity. Therefore, we used the regional dipole model in the present study. It would be interesting investigate dipole localization of training-induced alpha activity by different methods in future research.

Thanks for the reviewer’s constructive suggestion. You mentioned that we did not explore the memory function in this study. We agree and have corrected the description in the abstract (lines 2-3). In addition, we have also reorganized the discussion in the “Discussion” section (please see point 6).

(lines 2-3) *“**Much of the work in alpha NFT has focused on evaluating changes in alpha amplitude.”*

2. Many questions remain throughout the methods section that make me wonder whether the used methods were the most appropriate ones to answer the research question:

- What is the three-dipole model that was mentioned in the abstract but never explained in the manuscript?

**Response:** Thank you for the reviewer’s suggestion. No recent studies have provided information about the sources of training-induced alpha activity. In this study, we found that sources of training-induced alpha activity were mostly clustered in the precuneus, PCC and middle temporal gyrus. These three regions would be good candidates for dipole source localization, which could help researchers to decide on the number of dipoles in future studies. We have corrected “three-dipole model” to “three dipoles” in the abstract (lines 24).

- Was the neurofeedback training on fixed days or were the three sessions per week conducted randomly?

**Response:** Thank you for the reviewer’s suggestion. The neurofeedback training was performed on fixed days and at fixed times. The days and times were chosen by discussing them with the participants.

- Why was feedback to EEG activity over central sites given during neurofeedback training? On what basis were these electrodes chosen? What does the chosen neurofeedback protocol mean for other alpha neurofeedback studies that give feedback to other electrode sites?

**Response:** Previous alpha NFT studies have reported that alpha activity can be modulated using C3, Cz and C4 electrodes (Lecomte et al., 2011, https://doi.org/10.4236/psych.2011.28129; Nan et al., 2012, https://doi.org/10.1016/j.ijpsycho.2012.07.182.; Wei et al., 2017, https://doi.org/10.1186/s12938-017-0418-8.). In our previous study [1], we found that alpha activity was enhanced after NFT when electrodes located centrally (2.5 cm anterior and posterior to C3, Cz, and C4). The evidence indicated that these electrodes were good candidates for alpha NFT. Therefore, in this study, we used the same electrodes as in our previous study. Indeed, it would be interesting to investigate the effects of different electrodes on modulating alpha activity in future research.

- Why was resting-state EEG only investigated for responders? How many participants were responders?

**Response:** In this study, “responders” were defined as well-trained participants, meaning that they had learned a strategy to enhance their alpha activity successfully. Therefore, twenty-nine participants (83%) in the Alpha group were identified as well-trained “responders” and participated in whole-head EEG recording. The whole-head EEG recording contained fixation (same as the baseline block of neurofeedback training, during which participants were asked to keep their eyes open and not to engage in any training event from the training block) and alpha blocks (producing training-induced alpha activity). We used data of whole-head EEG to characterize the topographic map and dipole source localization of training-induced alpha activity.

- Did the study have a different aim during conduction – why would you need the control group then?

**Response:** We added the control group, which was compared to the alpha group to test the effect of alpha NFT on alpha enhancement. Both groups showed no significant differences in alpha amplitude at the beginning, indicating that the characteristics of the alpha group were the same as those of the control group. After NFT, the control group showed no change in alpha amplitude. In contrast, the alpha group showed significant differences in alpha amplitude compared to the control group. The results confirmed that our training protocol had effective results for alpha enhancement.

- Did participants use strategies during resting-state (p. 5, lines 103-105) and if so, why?

**Response:** No. In the resting period, we helped the participants recall the type of strategy that they used to achieve high alpha amplitude from the previous training block. Therefore, in this period, no EEG recording was conducted, and we only talked to the participants. Subsequently, the next training block was conducted.

- Was the frequency band for the control group different for every session or did it repeat by chance?

**Response:** No, there were no significant differences between sessions. ANOVA revealed no significant main effect of the session on delta (*p* = 0.179), theta (*p* = 0.108), alpha (*p* = 0.157), and beta (*p* = 0.104).

- What were the criteria for artifact removal (p. 6, line 163)? How were eye and muscle artifacts controlled for during neurofeedback training? How much data had to be removed for analysis?

**Response:** Thank you for the reviewer’s suggestion.

1. Successful alpha events contaminated by blinks, eye-movement artifacts (> 65 μV) or remarkable muscle activity were automatically removed (lines 184-185).

2. Before neurofeedback training, we asked the participants not to use inadequate strategies involving eye closure or body movements during training. In addition, we used a digital camera to exclude the effects of possible behavioral artifacts (lines 116-119). Bipolar recording was also beneficial to reduce the possible artifacts of motion or eye blinks. Therefore, rare data (< 1%) were removed for analysis.

- What did participants do during the 5-minute breaks of the whole-head EEG measurement?

**Response:** The participants took a break for 5 minutes without EEG measurements and waited for the next run.

- What does a positive / negative alpha peak (p.7, lines 171-178)? Would an alpha increase/decrease be a more fitting description?

**Response:** Thank you for the reviewer’s suggestion. We have corrected the description in the whole-head EEG and processing part of the “Materials and methods” section.

(lines *187-190) “**The time points of the highest voltage positive peak of the successful alpha events were identified and defined as trigger points. Based on the time points, 1.2-s alpha epochs centered on the positive peak of the artifact-free data were extracted and averaged.”*

*(lines 195-197) “If* *the first peak voltage in an alpha epoch was positive or negative, it was categorized as a positive alpha epoch or a negative alpha epoch, respectively.”*

- How many trials of the whole-head EEG measurement were used for each analysis method? Why did the number of trials differ for the four analysis methods (p. 16)?

**Response:** When a 1-s EEG alpha amplitude of the alpha blocks was higher than the 1.5-fold averaged alpha amplitude of all of the 1-s fixation EEGs, the 1-s EEG segment (epoch) was considered a successful alpha event (trials). For each “responder,” approximately 250-500 trials were used for analysis. All of the trials were used for the MPA and ERSPA. However, to identify whether the first peak in a trial was positive or negative, all of the trials were assigned to positive trials and negative trials. Therefore, the numbers of trials for PA and NA methods were smaller than for MPA and ERSPA methods.

3. Topographic results are reported only on a visual basis (p. 9)! Please report statistical analyses if you want to discuss differing topographies. Similarly, the source localization results were statistically compared between methods (pp. 10-11) but localized brain regions were not tested statistically against each other within the same method (e.g., is the source localization of the precuneus, PCC, and middle temporal gyrus detected by the positive average significantly greater than zero or greater than detection of other regions using the same method?).

**Response:** Thank you for the reviewer’s constructive suggestion. In this version, we have evaluated differences among the alpha amplitudes of frontal (FP1, FP2, F3, FZ, F4 FC3, FCZ and FC4), parietal (CP3, CPZ, CP4, P3, PZ and P4), occipital (O3, OZ and O4), frontotemporal (F7, F8, FT7 and FT8), and parietotemporal (TP7, TP8, T5 and T6) regions with each method (lines 207-211). The statistical results have been added in the EEG mapping of training-induced alpha activity part of the “Results” section (lines 294-323). The percentage of “responders” in each localized brain region are tested statistically against zero in each method. The results have been added to the dipole source localization part of the “Results” section (lines 342-355).

*(*lines 207-211*) “**Furthermore, to evaluate the alpha-dominant region, the alpha amplitudes of the averaged alpha epoch of the electrodes close to* *the frontal (FP1, FP2, F3, FZ, F4 FC3, FCZ and FC4), parietal (CP3, CPZ, CP4, P3, PZ and P4), occipital (O3, OZ and O4), frontotemporal (F7, F8, FT7 and FT8), and parietotemporal (TP7, TP8, T5 and T6)* *regions were averaged separately.”*

(lines 294-323) “*For the MPA method, the highest peak in the middle of the alpha epoch and a flat period on both sides were observed. Significant differences among the alpha amplitudes of the parietal and frontal (t = 2.137, p = 0.041), parietal and occipital (t = 6.558, p < 0.001), parietal and frontotemporal (t = 6.583, p < 0.001), parietal and parietotemporal (t = 7.335, p < 0.001), frontal and frontotemporal (t = 15.239, p < 0.001), frontal and parietotemporal (t = 2.315, p = 0.028), occipital and frontotemporal (t = 3.680, p = 0.001), and parietotemporal and frontotemporal (t = 3.640, p = 0.001) regions were observed. For the PA method, the largest positive peak followed the start of the alpha epoch (trigger point). Significant differences among the alpha amplitudes of the parietal and frontal (t = 2.340, p = 0.027), parietal and occipital (t = 5.699, p < 0.001), parietal and frontotemporal (t = 4.521, p < 0.001), parietal and parietotemporal (t = 4.555, p < 0.001), frontal and occipital (t = 1.793, p = 0.007), frontal and frontotemporal (t = 2.778, p = 0.010), occipital and frontotemporal (t = -0.784, p = 0.035), and occipital and parietotemporal (t = -1.055, p = 0.001) regions were observed. For the NA methods, the negative peak followed the start of the alpha epoch (trigger point). Significant differences among the alpha amplitudes of the parietal and frontal (t = 1.356, p = 0.020), parietal and occipital (t = 4.518, p < 0.001), parietal and frontotemporal (t = 4.424, p < 0.001), parietal and parietotemporal (t = 5.217, p < 0.001), frontal and occipital (t = 1.627, p = 0.040), frontal and frontotemporal (t = 5.969, p < 0.001), occipital and parietotemporal (t = -0.824, p = 0.032), and occipital and frontotemporal (t = 1.003, p = 0.042) regions were observed. Before the trigger point, a flat period was observed with the PA and NA methods. For the ERSPA method, alpha activity was obviously observed after inverse FFT implementation. Significant differences among the alpha amplitudes of the parietal and frontal (t = 0.714, p = 0.045), parietal and occipital (t = 1.077, p = 0.003), parietal and frontotemporal (t = 1.751, p = 0.002), parietal and parietotemporal (t = 2.568, p = 0.002), frontal and occipital (t = 0.622, p = 0.042), frontal and frontotemporal (t = 1.332, p = 0.049), occipital and parietotemporal (t = 0.299, p = 0.026), and parietotemporal and frontotemporal (t = 0.195, p = 0.028) regions were observed. These data indicated that training-induced alpha activity in the parietal region was significantly higher than other regions.”*

(lines 342-355) *“**For the MPA method, the percentages of “responders” whose dipoles were clustered in the precuneus, PCC and middle temporal areas were 84% (p < 0.001), 68% (p < 0.001) and 40% (p < 0.001) significantly different from zero, respectively. For the PA method, the percentages of “responders” whose dipoles were clustered in the precuneus, PCC and middle temporal areas were 72% (p < 0.001), 36% (p = 0.001) and 48% (p < 0.001) significantly different from zero, respectively. For the NA method, the percentages of “responders” whose dipoles were clustered in the precuneus, PCC and middle temporal areas were 64% (p < 0.001), 40% (p = 0.005) and 40% (p < 0.001) significantly different from zero, respectively. For the ERSPA method, the percentages of “responders” whose dipoles were clustered in the precuneus, PCC and middle temporal areas were 64% (p < 0.001), 32% (p = 0.002) and 44% (p < 0.001) significantly different from zero, respectively.* *The percentage of “responder” whose dipoles were located in the other regions was ≤ 16% for each method and showed no significant different from zero.”*

4. The source localization results reported in the results section (p. 10) differ from the results displayed in Table 1 (p. 11). The authors state that 32%-84% of the participants showed dipoles in the reported regions of the precuneus, PCC, and middle temporal gyrus. However, the results in the text are bilateral and the results in the table are split for left and right brain regions. How can you exclude that a participant showing a dipole in right precuneus didn’t also show a dipole in left precuneus? The authors simply added the number of participants for left and right dipoles together. Looking at the results split for left and right brain regions, in some regions barely showed a dipole (4%-16%, which corresponds to 1-4 participants).

**Response:** Thank you for the reviewer’s constructive suggestion. In this study, dipoles of each “responder” were located in different regions. The dipole locations were also different between “responders.” In this revised version, we have removed the term “bilateral” from the text. The number (N) of “responders” in Table 2 indicates the number of “responders” whose dipoles are located in the brain regions, whether in one or both hemispheres. For example, if one dipole is located in the right precuneus or one in the right and one in the left, the number (N) increased by 1.

5. Considering that 63 participants took part in the study in total, the source localization results rely on a very small number of participants (1-14 participants for the localized brain regions). What does that mean for the generalization of the results? I doubt that effect sizes (that were not reported) are considerably large.

**Response:** Thank you for the reviewer’s suggestion. In this study, only well-trained “responders” in the Alpha group participated in whole-head EEG recording (lines 260-262). The whole-head EEG data of twenty-five “responder” were used to perform dipole source localization. The number (N) of “responders” in Table 2 indicates how many “responders” whose dipoles were located in the brain regions, whether in one or both hemispheres. Therefore, the percentage of “responders” whose dipoles were clustered in the precuneus (64%-84%), PCC (36%-68%) and middle temporal areas (40%-48%) were significantly different from zero among the four methods (lines 342-355). The results indicated that the precuneus, PCC and middle temporal gyrus seem to play important roles in enhancing training-induced alpha activity.

6. The authors tried to integrate their results in the current literature in the discussion. However, it is not clear how the source localization in the present study specifically contributes to the understanding of alpha neurofeedback training. Specifically, the conclusion about a “global alpha activity” (p. 17) that spreads from parietal to frontal regions is highly speculative and is not supported by the results (sources in parietal regions solely). Furthermore, the interpretation that sources of alpha activity after neurofeedback training are regions important for memory because of the strategies that participants used lacks any evidential basis. If a conclusion about memory performance based on neurofeedback is drawn, the authors should systematically investigate transfer to memory tasks or explore the nature of used strategies during training and the following whole-head EEG measurement.

**Response:** **Response:** We agree and **t**hank you for the reviewer’s constructive suggestion. Previous alpha NFT studies reported that cognitive performance was improved after alpha NFT. These findings indicated that participants learned to self-regulate their brains to increase alpha activity, which could improve their cognitive performance. However, there are no studies investigating which regions of the brain work when enhancing alpha activity. If we can determine the sources of the training-induced alpha activity, it could provide valuable information to support cognitive improvement after alpha NFT. Indeed, we did not investigate the effect of alpha NFT on memory performance. In the revised version, we have reorganized and added an extensive discussion to the “Discussion” section (lines 468-489). Furthermore, we also have evaluated differences among the alpha amplitudes of the frontal, parietal, occipital, frontotemporal, and parietotemporal electrodes with each method. The results indicated that the training-induced alpha activity of the parietal region was significantly higher than in the other regions. We have reorganized this discussion in the “Discussion” section (lines 490-507).

(lines 468-489) *“**Alpha activity has been thought to be associated with attention function. For example, alpha power increases were found during internal attention tasks [30] and decreases during demanding arithmetic tasks [31]. In the present study, participants learned to self-regulate alpha activity by shifting internal (mental imagery) and external attention (feedback) during training. Such a shift produces comparable signatures of alpha activity modulations [32]. Alpha activity increases reflected internal attention of mental imagery [33]. In neuroimaging studies, the precuneus is involved in visuospatial attention functions [34]. The PCC regulates the focus of attention and controls the balance between internal and external attention [35]. Lesions of the middle temporal gyrus were associated with spatial attention deficits in spatial neglect patients [36]. All of the evidence supports the existence of a range of attention-related influences on activation in the precuneus, PCC and middle temporal gyrus. The amplitude of alpha increases reflects cortical inhibition [37]. Inhibition of these three brain regions could protect reorienting against irrelevant stimulation, which could modulate attention function more sufficiently. Consequently, after training, participants might efficiently modulate internal attention to increase alpha activity. Internal attention involves modulation, selection, and maintenance of internally produced information, such as long-term memory or working memory [38]. Considered together, alpha NFT could potentially modulate these three attention-related regions to improve internal attention function and further affect memory performance. Our findings could provide valuable information to* *partially support memory improvement after alpha NFT in previous studies.”*

(lines 490-507) “*In the present study, a larger alpha amplitude was observed in the parietal region than in other regions, indicating that training-induced alpha activity dominated in the parietal region. The result was similar with the dipole locations (the PCC and precuneus). In addition, alpha activity also appeared slightly higher in the frontal region. Alpha NFT was thought to modulate top-down control networks, resulting in participants learning successfully to self-regulate their alpha activity. An increased frontal alpha amplitude might enable tight functional coupling between frontal cortical areas, thereby allowing for control of visual processing [39]. Alpha oscillations of frontal brain areas seem to be important for top-down control [4041]. A recent simultaneous EEG-fMRI study further found that long-range alpha synchrony was intrinsically linked to activity in the frontal-parietal control network [42]. This evidence could support frontal alpha activity being slightly enhanced by alpha NFT. Another possible reason for increased frontal alpha amplitudes might be “neural efficiency.” NFT could cause the neurons of the frontal region to integrate information over space and time repeatedly, causing* *“neural efficiency” [43]. Neural efficiency hypothesizes that neural activation is decreased in experts. Well-trained “responders” as experts had learned to decrease neural activation of frontal region after NFT, resulting in increased frontal alpha activity.”*

**Reviewer #3**: Dear authors,

I am sorry to inform you that I did not recommend your study for publication.

I realize that you performed an extensive study with 12 sessions of neurofeedback training and your neurofeedback data looks very promising (although you should use an identical instruction for the experimental and the control group, to make sure the alpha increase originates from NFT and not from your instruction to think of positive memories (which in itself leads to relaxation and to increased alpha)).

I also really liked your camera installation during neurofeedback training, that is a great method!

The main concern I have with your study is the attempt to perform EEG source localization with an 32 electrode EEG cap. Here is a paper you may want to look at (https://www.sciencedirect.com/science/article/pii/S0165027015003064), where EEG data was simulated and the validity of source localization with different electrode densities was tested. Everything below 64 electrodes was simply inaccurate, but even 64 electrodes wasn't good. It should not be performed with anything below 128 electrodes (and even then usually T1 scans of each participant are used).

**Response:** Thank you for the reviewer’s suggestion. Indeed, source localization accuracy is improved when electrode numbers increase. Source localization with EEG is used to estimate approximate brain regions. Rodin and Rodin (1995) reported a satisfactory result when sources of averaged occipital alpha epochs were located over the occipital region using 19 electrodes. Sohrabpour et al. (2015) (https://doi.org/10.1016/j.clinph.2014.05.038) reported that dipoles of each single epileptic spike clustered in similar brain regions when electrode numbers decreased from 128 to 96, 64 and 32. Based on the concept of these findings, we averaged alpha epochs to increase the SNR for each participant but did not the average alpha epochs of all “responders”. After dipole source localization, we found dipoles approximately clustered in the bilateral precuneus, PCC and middle temporal regions. Certainly, different brain activity, head models, source localization methods, electrode numbers and SNRs will affect the accuracy of source localization. For training-induced alpha activity, these issues will be interesting for future research.

If you want to use your dataset for future publications, please also make sure you do present correct units in every figure and also do not mix up amplitude and power in the manuscript.

**Response:** Thank for reviewer’s suggestion. In the present study, we did not transform the amplitude of FFT, for example, square the amplitude as power. Therefore, the units are the same as the original signal unit (microvolt).

Best wishes and all the best

Marion Brickwedde

**Reviewer #4**: The present study aimed to identify sources of training-induced alpha activity through four different temporal/spectral analytic techniques, relating the effects of the training to ”memory function”.

As I understood, the authors have already reviewed their paper. In my opinion, the authors did a very good work, I also guess thanks to the valuable reviewers' feedback.

The paper now is very well structured, with a properly deep introduction and large and detailed discussions.

Undoubtedly, I believe some weaknesses still affect the work, most of all the generic reference of alpha power in their study to memory functions without directly testing this reference by means of a specific memory task used in memory studies carried out in the context of cognitive neuroscience literature on this matter.

Indeed, I was most surprised of finding this reference in the light of the kind of procedure to whom their experimental participants were submitted, namely a neurofeedback training (NFT) to self-regulate their own brain activity. Indeed, rather than a mnemonic task, this can be referred to as a learning to self-regulate their own brain alpha activity by means of a progressive increase of individual cognitive control on brain neural networks subserving alertness and focusing of attention functions.

However, I believe that this may not be hard to solve now. Indeed, I think that the hard work made so far by the authors would deserve one more chance to achieve an acceptable level and be published.

Indeed, the EEG literature is plenty of studies directly relating alpha power to the functions of alerting, orienting, and cognitive control characterizing the different neural networks of the visual selective attention system. Below, for instance, I reported a short list of recent and less recent studies in the literature on alpha power and visual attention to be used as a starting point for a critical review of their findings and of those papers referred to by these studies:

Zani, A.; Tumminelli, C.; Proverbio, A.M. Electroencephalogram (EEG) Alpha Power as a Marker of Visuospatial Attention Orienting and Suppression in Normoxia and Hypoxia. An Exploratory Study. Brain Sci. 2020, 10, 140.

Misselhorn, J.; Friese, U.; Engel, A.K. Frontal and parietal alpha oscillations reflect attentional modulation of cross-modal matching. Sci. Rep. 2019, 22, 5030.

Rihs, T.A.; Michel, C.M.; Thut, G. Mechanisms of selective inhibition in visual spatial attention are indexed by alpha-band EEG synchronization. Eur. J. Neurosci. 2017, 25, 603–610.

Poch, C.; Carretie, L.; Campo, P. A dual mechanism underlying alpha lateralization in attentional orienting to mental representation. Biol. Psychol. 2017, 28, 63–70.

Foxe, J.J.; Snyder, A.C. The role of alpha-band brain oscillations as a sensory suppression mechanism during selective attention. Frnt. Hum. Neurosci. 2011, 2, 154.

Kelly, S.P.; Lalor, E.C.; Reilly, R.B.; Foxe, J.J. Increases in Alpha Oscillatory Power Reflect a Suppression during Sustained Visuospatial Attention Active Retinotopic Mechanism for Distracter. J. Neurophysiol. 2006, 95, 3844–3851.

As I already wrote, I believe that if the authors will be able to eliminate any reference to generic and abstract “memory functions” both in the Abstract and in any other paper sections, mostly the Introduction and Discussion sections, and to provide a short critical discussion of their NFT alpha findings in the light of the attentional findings advanced in the indicated studies, the paper will be more than worth to be published.

**Response:** Thank you for the reviewer’s constructive suggestion. Indeed, participants learned to self-regulate their own brain alpha activity by shifting internal (mental imagery) and external attention (feedback) during training. Progressively, attention function can increase through NFT. Attention and memory are thought to interact closely. From this point of view, it could provide valuable information to support memory improvement after alpha NFT in previous studies. An extensive discussion has been added to the “Discussion” section.

(lines 468-489) *“*Alpha activity has been thought to be associated with attention function. For example, alpha power increases were found during internal attention tasks [30] and decreases during demanding arithmetic tasks [31]. In the present study, participants learned to self-regulate alpha activity by shifting internal (mental imagery) and external attention (feedback) during training. Such a shift produces comparable signatures of alpha activity modulations [32]. Alpha activity increases reflected internal attention of mental imagery [33]. In neuroimaging studies, the precuneus is involved in visuospatial attention functions [34]. The PCC regulates the focus of attention and controls the balance between internal and external attention [35]. Lesions of the middle temporal gyrus were associated with spatial attention deficits in spatial neglect patients [36]. All of the evidence supports the existence of a range of attention-related influences on activation in the precuneus, PCC and middle temporal gyrus. The amplitude of alpha increases reflects cortical inhibition [37]. Inhibition of these three brain regions could protect reorienting against irrelevant stimulation, which could modulate attention function more sufficiently. Consequently, after training, participants might efficiently modulate internal attention to increase alpha activity. Internal attention involves modulation, selection, and maintenance of internally produced information, such as long-term memory or working memory [38]. Considered together, alpha NFT could potentially modulate these three attention-related regions to improve internal attention function and further affect memory performance. Our findings could provide valuable information to partially support memory improvement after alpha NFT in previous studies.*”*

As a more specific point, please provide a very short explanation of the NFT procedure already in the Abstract.

**Response:** Thank you for the reviewer’s suggestion. We have provided an explanation of the NFT procedure in the abstract.

(lines 7-16) *“**Thirty-five healthy participants were recruited into an alpha group receiving feedback of 8–12-Hz amplitudes, and twenty-eight healthy participants were recruited into a control group receiving feedback of random 4-Hz amplitudes from the range of 7 to 20 Hz. Twelve sessions were performed within 4 weeks (3 sessions per week). The control group had no change in alpha amplitude. In contrast, twenty-nine participants in the alpha group showed significant alpha amplitude increases and were identified as “responders.” A whole-head EEG was recorded for the “responders” after NFT. The epochs of training-induced alpha activity from whole-head EEG were averaged by four different methods for equivalent current dipole source analysis.”*

6. PLOS authors have the option to publish the peer review history of their article ([what does this mean?](https://journals.plos.org/plosone/s/editorial-and-peer-review-process#loc-peer-review-history)). If published, this will include your full peer review and any attached files.

If you choose “no”, your identity will remain anonymous but your review may still be made public.

**Do you want your identity to be public for this peer review?** For information about this choice, including consent withdrawal, please see our [Privacy Policy](https://www.plos.org/privacy-policy).

Reviewer #1: No

Reviewer #2: No

Reviewer #3: **Yes:** Marion Brickwedde, PhD

Reviewer #4: No

[NOTE: If reviewer comments were submitted as an attachment file, they will be attached to this email and accessible via the submission site. Please log into your account, locate the manuscript record, and check for the action link "View Attachments". If this link does not appear, there are no attachment files.]

While revising your submission, please upload your figure files to the Preflight Analysis and Conversion Engine (PACE) digital diagnostic tool, <https://pacev2.apexcovantage.com/>. PACE helps ensure that figures meet PLOS requirements. To use PACE, you must first register as a user. Registration is free. Then, login and navigate to the UPLOAD tab, where you will find detailed instructions on how to use the tool. If you encounter any issues or have any questions when using PACE, please email PLOS at [figures@plos.org](mailto:figures@plos.org). Please note that Supporting Information files do not need this step.
