# Supplementary material for: Equivalent current dipole sources of neurofeedback training-induced alpha activity through temporal/spectral analytic techniques
Source: PLoS One. 2022 Feb 25;17(2):e0264415. doi: 10.1371/journal.pone.0264415 (PMC8880644; doi:10.1371/journal.pone.0264415)
Supplement: Attachment — Submitted filename: Response to Reviewers2.docx [file pone.0264415.s004.docx]

Dear Dr. Shaw,

Thank you for submitting your manuscript to PLOS ONE. After careful consideration, we feel that it has merit but does not fully meet PLOS ONE’s publication criteria as it currently stands. Therefore, we invite you to submit a revised version of the manuscript that addresses all the points raised during the review process.

In particular, I require that you provide full analyses including the non-responders.

**Response:** Thank you for the editor’s suggestion. In this study, NFT was conducted for all (sixty-three) participants. According to the criteria of “responder” (lines 167-169), six non-well-trained participants (“non-responders”) of the Alpha group were identified and excluded from further analyses, e. g., whole-head EEG data, EEG mapping and dipole source localization. In other words, only well-trained “responders” in the Alpha group participated in whole-head EEG recording, but not “non-responders”. Therefore, there are no analyses of whole-head EEG data, EEG mapping and dipole source localization for “non-responders”. In this revised version, we have added the comparison of the Ctrl group to the all participants of the Alpha groups in alpha amplitude (lines 267-275) and the amplitudes of bands (lines 284-293). The statistics have been added in the EEG data for NFT part of the “Results” section. The analysis of the alpha amplitude and successful alpha events between the fixation and alpha blocks were also added in the “Whole-head EEG data” part of the “Results” section (lines 303-311).

Reviewer #1:

Dear Authors,

I still have some concerns related to your manuscript:

1. In my opinion only comparison of the whole control group to the all members of the experimental group is the prove of successful intervention, Does Fig 1 show all members of alpha group or only responders?

**Response:** Thank you for the reviewer’s suggestion. The Fig 1 and Fig 2 show only responders of the Alpha group in the previous version. In this version, we have changed Fig 1 and Fig 2 which show all members of the Alpha (thirty-five participants) and Ctrl groups. Besides, the statistics have been added in the EEG data for NFT part of the “Results” section.

(lines 267-275) *“The mean relative alpha amplitude progressively increased throughout the 12 sessions in the Alpha group but not in the Ctrl group (Fig. 1). ANOVA revealed significant main effects of group (F_1,61_ = 16.382, p < 0.001), session (**F_11,671_ = 5.726, p < 0.001), and their interaction (**F_11,671_ = 4.642, p < 0.001). The mean relative alpha amplitude showed no significant difference in the Ctrl group throughout NFT. In contrast, the mean relative alpha amplitudes of the 5th-12th sessions in the Alpha group significantly differed from those of the 1st session. In addition, a linear increase was observed in the Alpha group (R ^2^ = 0. 103, p < 0.001).”*

(lines 284-293) *“For the delta, theta and beta bands, ANOVA revealed no significant main effect of group (delta, F1,61 = 3.281, p = 0.075; theta, F1,61 = 2.465, p = 0.122; beta, F1,61 = 0.644, p = 0.425), session (delta, F1,61 = 1.427, p = 0.237; theta, F1,61 = 3.228, p = 0.077; beta, F1,61 = 1.166, p = 0.285), and their interaction (delta, F1,61 = 0.002, p = 0.966; theta, F1,61 = 0.230, p = 0.634; beta, F1,61 = 0.488, p = 0.488). In contrast, for the alpha band, significant main effects of group (F1,61 = 4.535, p = 0.037), session (F1,61 = 20.530, p < 0.001), and their interaction (F1,61 = 4.466, p = 0.039). The alpha band showed a significant difference between the two groups in the 12th session, but not in the 1st session. A significant difference between the 1st and 12th sessions in the alpha group was also observed.”*

2. There is no point to Investigate whole brain EEG of responders if you do not compare it to non-responders, it does not explain the difference between the two

**Response:** Thank you for the reviewer’s suggestion. “Responders” could successfully induce alpha activity to provide stable and reliable alpha activity, at least more effectively than “non-responders”. In other words, “responders” could provide more successful alpha epoch than “non-responders” which is helpful for the dipole source analyze. Therefore, only “responders” participated in whole-head EEG recording, but not “non-responders”. In order to reveal that “responder” had learned to enhance training-induce alpha activity, in this revise version, we have added the analysis of the alpha amplitude and successful alpha events between the fixation and alpha blocks. The results showed that both parameters were significant different (lines 300-306) indicated that “responder” had learned how to control and enhance alpha activity after NFT. The purpose of this study is to investigate the sources of training-induced alpha activity. Therefore, we did not compare and explain the difference between the two. This is another interesting issue for future NFT research.

(lines 303-309) “*The alpha amplitude of the alpha (13.02 ± 1.01) and fixation (10.01 ± 0.71) blocks were significant different (t = 4.822, p < 0.001). Besides, a significant difference between successful alpha events of the alpha (324.24 ± 3.97, range: 224-606) and fixation blocks (55.03 ± 22.27, range: 11-89) was also observed (t = 12.53, p < 0.001). These data indicated that “responders” had learned to successfully induce alpha activity in the alpha block. The successful alpha events were further processed through four different averaging methods.*”

3. In the questions about other bands I was asking about possible training effects - so to say to compare them between control and experimental groups

**Response:** Thank you for the reviewer’s suggestion. For the delta, theta and beta bands, no significant difference was observed between the Ctrl and Alpha groups, and 1st and 12th sessions. In contrast, for the alpha band, significant differences were observed between the two groups in the 12th session, and between the 1st and 12th sessions in the Alpha group. The results were the same as the previous results indicated that only the amplitude of alpha band was increased after NFT but not the other bands. The detail statistics have been added in the EEG data for NFT part of the “Results” section.

(lines 284-293) *“For the delta, theta and beta bands, ANOVA revealed no significant main effect of group (delta, F1,61 = 3.281, p = 0.075; theta, F1,61 = 2.465, p = 0.122; beta, F1,61 = 0.644, p = 0.425), session (delta, F1,61 = 1.427, p = 0.237; theta, F1,61 = 3.228, p = 0.077; beta, F1,61 = 1.166, p = 0.285), and their interaction (delta, F1,61 = 0.002, p = 0.966; theta, F1,61 = 0.230, p = 0.634; beta, F1,61 = 0.488, p = 0.488). In contrast, for the alpha band, significant main effects of group (F1,61 = 4.535, p = 0.037), session (F1,61 = 20.530, p < 0.001), and their interaction (F1,61 = 4.466, p = 0.039). The alpha band showed a significant difference between the two groups in the 12th session, but not in the 1st session. A significant difference between the 1st and 12th sessions in the alpha group was also observed.”*

4. 4. All methodological explanations should be included in methods section

**Response:** Thank you for reviewer’s suggestion. We have added the methodological explanations in the “Methods” section, e.g., the description of the trainer (lines 113-115), the benefit of the bipolar recording (lines 131-132), the 3D digitizer for individual electrode positions (lines 174-175), the calculation of ERSPA (lines 205-208) and the ICA for dipole source localization (lines 221-228).

Reviewer #3:

The authors used a 12-session alpha Neurofeedback training to induce high-alpha activity in a group of responders. These individuals underwent a whole-head EEG recording and were asked to emulate the strategy used during neurofeedback training in order to produce high alpha activity. During this time, high alpha bursts were used to apply EEG source localisation to find sources of NF-training induced alpha power changes. I believe that the study design underlies some major confounds and that this conclusion cannot be drawn so easily and that these limitations must be clearly addressed in the discussion.

1. Systematic differences in study design between groups, unrelated to the condition differences
a. Alpha group received different instructions than control group (mental imagery, etc.)

**Response:** Thank you for the reviewer’s suggestion. Both Ctrl and Alpha groups received the same instructions.

b. Alpha group received verbal feedback of successful alpha events (verbal feedback has a strong motivational effect, which can strongly influence learning processes). In case the control group received the same verbal feedback for successful events in their frequency band, this point can be ignored.

**Response:** Thank you for the reviewer’s suggestion. Both Ctrl and Alpha groups received same verbal feedback.

c. “not well trained” participants were removed from alpha group, but no such treatment was applied to the control group. How many participants of the control group would have to be excluded applying the same criteria? --> This makes it impossible to conclude, that found differences between groups originate from NF training.

**Response:** Thank you for the reviewer’s suggestion. In this study, NFT was conducted for all (sixty-three) participants we recruited. According to the criteria of “responder” (lines 167-169), six non-well-trained participants of the Alpha group were identified and excluded from further analyses, e. g., whole-head EEG data, EEG mapping and dipole source localization. We have modified the description to the last paragraph of the “EEG data for NFT” part of the “Results” section.

(lines 299-301) *“Twenty-nine participants (83%) were identified as “responders” in this study. Therefore, six participants in the Alpha group who were not well trained were excluded from whole-head EEG data, EEG mapping and dipole source localization analyses.”*

2. There is no proof that the localization of alpha power is related to NF training.
a. There is no comparison between high alpha burst events in a baseline period before any training happened concerning their dipoles reconstructions and successful alpha events after NF training. It is entirely possible they would be the same and that NF training simply increases already present alpha oscillations, targeted by electrodes over the training site.

Response: Thank you for the reviewer’s constructive suggestion. The purpose of NFT is to enable an individual to learn to self-regulate specific frequency band(s) which already exist in brain activity. Therefore, it is possible that NFT simply increases already present alpha oscillations. In this study, the Alpha group showed successful training of the alpha activity after NFT, but not the Ctrl group. In addition, in order to reveal that participants had learned to enhance training-induce alpha activity, the whole-head EEG was recorded after NFT. The results showed that the alpha amplitude and successful alpha events between the fixation and alpha blocks were significant different indicated that participants had learned how to control and enhance alpha activity after NFT. These results have been added in the “Whole-head EEG data” part of the “Results” section.

(lines 303-309) “*The alpha amplitude of the alpha (13.02 ± 1.01) and fixation (10.01 ± 0.71) blocks were significant different (t = 4.822, p < 0.001). Besides, a significant difference between successful alpha events of the alpha (324.24 ± 3.97, range: 224-606) and fixation blocks (55.03 ± 22.27, range: 11-89) was also observed (t = 12.53, p < 0.001). These data indicated that “responders” had learned to successfully induce alpha activity in the alpha block. The successful alpha events were further processed through four different averaging methods.*”

b. The authors did not present a comparison between the fixation period of the whole-head EEG and the periods, where participants had to copy their NF strategies. If there is no difference overall in alpha power between both periods, how can be inferred that this alpha activity has anything to do with NF training? What if the same source localisation strategy would be applied to high alpha bursts in the fixation period? If it was different, that would be more convincing. How many successful alpha events would there be in the fixation period, if the same criteria (1.5-fold higher amplitude than average of all the 1-s fixation EEGs) was applied?

Response: Thank you for the reviewer’s constructive suggestion. In this revised version, we have compared the alpha amplitude and successful alpha events between the fixation and alpha blocks. Both parameters showed significant differences between the alpha and fixation blocks. These results have been added in the “Whole-head EEG data” part of the “Results” section.

(lines 303-309) *“The alpha amplitude of the alpha (13.02 ± 1.01) and fixation (10.01 ± 0.71) blocks were significant different (t = 4.822, p < 0.001). Besides, a significant difference between successful alpha events of the alpha (324.24 ± 3.97, range: 224-606) and fixation blocks (55.03 ± 22.27, range: 11-89) was also observed (t = 12.53, p < 0.001). These data indicated that “responders” had learned to successfully induce alpha activity in the alpha block. The successful alpha events were further processed through four different averaging methods.”*

3. The source localisation cannot be generalised for all NF alpha trainings
a. Alpha power reported in previous studies, generally increased over sites it was trained on. In this study, a general inference is made of where training-induced alpha changes occur, but it is evident that this is heavily dependent on training site.

Response: Thank you for the reviewer’s constructive suggestion. We have modified the description in the “Discussion” section.

(lines 486-488) “*our findings provide valuable information for future studies that three dipoles could be used for dipole source analysis of training-induced alpha activity, especially the training sites are around the central regions.*”

4. Insufficient electrode numbers and missing T1 scans introduce the possibility of heavy mis-localization errors (up to 7 cm). It is necessary that each of these limitations are clearly communicated in the discussion.

Response: Thank you for the reviewer’s suggestion. The low-density EEG used in dipole source localization may cause blurring and localization error. However, low-density electrodes used in dipole source localization may obtain valuable insight in application with some brain activities, such as epileptic spikes. Although the amplitude of alpha activity may not greater than that of epileptic activity, we used average method to increase SNR of alpha activity. A good SNR can decrease the estimation error of dipole source reconstruction. Besides, Fuchs et al. (2002) (https://doi.org/10.1016/S1388*-*2457(02)00030-5) reported that no significant differences in source localization were observed and showed the similarity in source localization between individual and standardized BEM models. The evidence revealed that standardized BEM models we used in this study may remain sufficient for source localization. A provided source solution remains an estimation that depends on priori assumptions which can be neurophysiological, biophysical and anatomical knowledge about the distribution of neuronal activity. However, there are still no assumptions about training-induced alpha activity to valid our findings. In the present study, we found dipoles approximately clustered in the bilateral precuneus, PCC and middle temporal regions. Of course, the validation of these findings should be tested in the future work, e.g., fMRI. We have modified the discussion of limitations in the “Discussion” section.

(lines 529-542) “*One of the limitations of the present study was low-density EEG. The low-density EEG used in dipole source localization may cause blurring and localization error. However, low-density EEG (<32 electrodes) used in dipole source localization may obtain valuable insight in application with some brain activities, such as epileptic spikes [44]. Although the amplitude of alpha activity may not greater than that of epileptic activity, we used average method to increase SNR of alpha activity. A good SNR can decrease the estimation error of dipole source localization. Indeed, more electrodes could reduce more distance errors in dipole source analysis [45]. More electrodes, such as 64 electrodes or more, should be tested in future research. The other limitation was absence of individual anatomical images. Individual anatomical image can improve the source localization accuracy. However, no significant differences in source localization were observed and showed the similarity in source localization between individual and standardized BEM models [46]. The evidence revealed that standardized BEM models used in this study may remain sufficient.*”

Some further details that need adjustment are outlined below

1. Previous studies have shown that training-induced alpha activity appeared diversely over the parieto-occipital [2], fronto-parietal [3], or frontal regions [4] through topographic EEG analysis … Therefore, estimating the properties of the internal localized sources of the topography was a valuable way to explore generators of training-induced alpha activity.

The first two studies only show that alpha power increased over exactly the sites that were trained on (the first study used P3, P4, Pz, O1 and O2 as training sites, and the second study used F3, Fz, F4, P3, Pz and P4 as training sites). In the third study the authors cite, alpha power increase was trained over POz in 2 participants and was successful over POz. In three additional participants, the frequency of alpha oscillations was trained to be sped up. These 3 participants showed increased frequency of alpha oscillations over frontal areas, that means a faster alpha rhythm. This did not refer to power changes.

Therefore, it is difficult to argue that in this study, the authors explore the generators of training-induced alpha activity. These studies clearly show that training-induced alpha power changes depend on training site. The authors can therefore only conclude results for the specified training site.

Response: Thank you for the reviewer’s constructive suggestion. We have modified the description in the “Discussion” section.

(lines 486-488) “*our findings provide valuable information for future studies that three dipoles could be used for dipole source analysis of training-induced alpha activity, especially the training sites are around the central regions.*”

2. Before NFT, researchers provided constructive strategies for successful alpha activity training reported by participants in our previous study, for example, pleasant or relaxing situations such as reading or wandering

this is a confound which needs to be discussed. This means that the instructions between conditions was not equal. It means that these strategies could have led to alpha increase independent of Neurofeedback training or EEG. It would only be no problem, if the control group received the same instructions.

Response: Thank you for the reviewer’s suggestion. Both Ctrl and Alpha groups received the same instructions.

3. In the resting period, the researchers use information on the cumulative waveform, e.g., the timestamps of high EEG amplitudes to help participants recall what kind of strategy they used to achieve a high amplitude.

was the same feedback given for the control group? Verbal feedback can have a tremendously motivating and positive effect on learning, which again is a confound for the NF training compared to control.

Response: Thank you for the reviewer’s suggestion. Both Ctrl and Alpha groups received the same verbal feedback in the resting period.

4. The 4-Hz bandwidth was randomly selected from the range of 7-20 Hz. Thus, the Ctrl group received various kinds of 4-Hz amplitudes during each session [16].

how many participants in the control group received feedback including 10 Hz in their training range?

Response: Thank you for the reviewer’s suggestion. The Ctrl group received amplitude of 4-Hz bandwidth as feedback. The 4-Hz bandwidth was randomly selected second-by-second from the range of 7-20 Hz during training block. In other words, the 4-Hz bandwidth was different between each second during training block for each participants of the Ctrl group. Therefore, all participants of the Ctrl group were possibly to receive feedback including 10 Hz. We have modified the description in the “Neurofeedback training and processing” part of the “Materials and methods” section.

(lines 144-146) “*The 4-Hz bandwidth was randomly selected second-by-second from the range of 7-20 Hz. Thus, the Ctrl group received various kinds of 4-Hz amplitudes during each training block.*”

5. The experimental procedure of the whole-head EEG recording involved 5 runs. Each run interleaved four fixation and four alpha blocks (50 s each). In a fixation block, the “responder” was asked to keep his or her eyes open and not to engage in any training event. In an alpha block, the “responder” was asked to produce training-induced alpha activity. There was a 5-min rest period between two consecutive runs.

As noted above, I find it important to report, whether the fixation and the alpha increase blocks different from each other in alpha power overall (not just looking at successful alpha events). If not, can the authors be certain their findings refer to the dipoles of NF training induced alpha power changes? Or just high alpha power events (which also happen naturally). Is there here found dipole localization any different to what has been found for resting state alpha oscillations?

Response: Thank you for the reviewer’s suggestion. In this revised version, we have compared the alpha amplitude and successful alpha events between the fixation and alpha blocks. Both parameters showed significant differences between the alpha and fixation blocks. These results have been added in the “Whole-head EEG data” part of the “Results” section.

(lines 303-311) “*The alpha amplitude of the alpha (13.02 ± 1.01) and fixation blocks (10.01 ± 0.71) were significant different (t = 4.822, p < 0.001). Besides, a significant difference between successful alpha events of the alpha (324.24 ± 3.97, range: 224-606) and fixation blocks (55.03 ± 22.27, range: 11-89) was also observed (t = 12.53, p < 0.001). These data indicated that “responders” had learned to successfully induce alpha activity in the alpha block. The successful alpha events were further processed through four different averaging methods.*”

6. All of the successful alpha events contaminated with blinks, eye-movement artifacts (> 65 μV) or remarkable muscle activity were automatically removed and were further processed through four different averaging methods.

I assume that the authors mean the remaining events were further processed, because it sounds as if the removed ones were processed.

Response: Thank you for the reviewer’s suggestion. We have corrected the description in the “Whole-head EEG and processing” part of the “Materials and methods” section.

(lines 188-192) *“The successful alpha events contaminated with blinks, eye-movement artifacts (> 65 μV) or remarkable muscle activity were automatically removed. After that, all of the remaining successful alpha events were further processed through four different averaging methods.”*

7. Six participants in the Alpha group who were not well trained were excluded from further analyses.

please state the exact exclusion criteria. Not well trained is very vague. If the authors would apply the same criterium to the ctrl group, how many participants would be excluded?

Response: Thank you for the reviewer’s suggestion. In this study, NFT was conducted for all (sixty-three) participants. According to the criteria of “responder” (lines 167-169), six non-well-trained participants of the Alpha group were identified and excluded from further analyses, e. g., whole-head EEG data, EEG mapping and dipole source localization. No participant of the Ctrl group was identified as “responder”. We have modified the description to the last paragraph of the EEG data for NFT part of the “Results” section.

(lines 299-301) *“Twenty-nine participants (83%) were identified as “responders” in this study. Therefore, six participants in the Alpha group who were not well trained were excluded from whole-head EEG data, EEG mapping and dipole source localization analyses.”*

8. How many trials were average for each method? Please state the min, mean and max in the text.

Response: Thank you for the reviewer’s suggestion. We have added the statistic in the “Whole-head EEG data” part of the “Results” section.

(lines 309-311) “*Finally, alpha epochs of the MPA and ERSPA (324.24 ± 22.27, range: 224-606), PA (186.03 ± 14.27, range: 66-358) and NA (138.21 ± 10.18, rang: 61-285) methods were identified and averaged.*”

9. The electrode number is still a problem and should be discussed. The authors refer to the paper from Rodin & Rodin, 1995, where a source localization was conducted with 19 electrodes. There is no proof in this paper, that this source localization was valid.

The second paper that is referred to by Sohrapour refers to the effect of electrode number on epileptic source localisation. Importantly, epileptic activity cannot be compared to healthy brain activity, as the strength of the activity largely exceeds normal activity, reducing error margin of source localization. Additionally, the patients studied in this particular study had seizures that strong, intracranial electrodes were implanted and surgery was conducted. Furthermore, in this study MRI scans of the individual participants were applied (which is not the case in the study under review). Also, the Sohrapour et. al find similar results for the source localisation, however the worst localisation is achieved using 32 electrodes. Excerpt from the paper: “In other words, the most dramatic decrease in localization error can be seen when going from 32 electrodes to 64 electrodes. The average localization error improves by 4 mm when going from 32 electrodes to 64 electrodes and improves by 1.3 mm when going from 64 electrodes to 96 electrodes,…Additionally to the computer simulation I cited before, I also want to refer to a review paper, elaborating on EEG source imaging by Michel & Brunet (https://doi.org/10.3389/fneur.2019.00325):“What is the minimal number of electrodes needed for reliable source localization? This question is often asked, particularly from the clinical community that intends to apply EEG source localization to the EEG that is routinely recorded with the standard 10-20 system, i.e., with only 19 electrodes. Several studies have demonstrated that this low number not only leads to blurring of the solution, but also to incorrect localization (49) compared the effective spatial resolution of different electrode montages (19-129 electrodes) and concluded that “the smallest topographic feature that can be resolved accurately by a 32-channel array is 7 cm in diameter, or about the size of a lobe of the brain.” All of this refers to source localisation in accordance with T1 scans.

Therefore, I believe it is not enough to state that the signal to noise ratio could be improved using T1 scans and higher density EEG, but to discuss as a limitation of the study, that the low-density EEG used introduces blurring and localization error, which in some cases can be substantial.

Response: Thank you for the reviewer’s suggestion. The low-density EEG used in dipole source localization may cause blurring and localization error. However, low-density electrodes used in dipole source localization may obtain valuable insight in application with some brain activities, such as epileptic spikes. Although the amplitude of alpha activity may not greater than that of epileptic activity, we used average method to increase SNR of alpha activity. A good SNR can decrease the estimation error of dipole source reconstruction. Besides, Fuchs et al. (2002) (https://doi.org*/*10.1016*/*S1388*-*2457(02)00030-5) reported that no significant differences in source localization were observed and showed the similarity in source localization between individual and standardized BEM models. The evidence revealed that standardized BEM models we used in this study may remain sufficient for source localization. The provided source solution remains an estimation that depends on priori assumptions which can be neurophysiological, biophysical and anatomical knowledge about the distribution of neuronal activity. However, there are still no assumptions about training-induced alpha activity to valid our findings. In the present study, we found dipoles approximately clustered in the bilateral precuneus, PCC and middle temporal regions. Of course, the validation of these findings should be tested in the future work, e.g., fMRI. We have modified the discussion of limitations in the “Discussion” section.

(lines 529-542) “*One of the limitations of the present study was low-density EEG. The low-density EEG used in dipole source localization may cause blurring and localization error. However, low-density EEG (<32 electrodes) used in dipole source localization may obtain valuable insight in application with some brain activities, such as epileptic spikes [44]. Although the amplitude* *of alpha activity may not greater than that of epileptic activity, we used average method to increase SNR of alpha activity. A good SNR can decrease the estimation error of dipole source localization. Indeed, more electrodes could reduce more distance errors in dipole source analysis [45]. More electrodes, such as 64 electrodes or more, should be tested in future research. The other limitation was absence of individual anatomical images. Individual anatomical image can improve the source localization accuracy. However, no significant differences in source localization were observed and showed the similarity in source localization between individual and standardized BEM models [46]. The evidence revealed that standardized BEM models used in this study may remain sufficient.*”
