# Supplementary material for: Equivalent current dipole sources of neurofeedback training-induced alpha activity through temporal/spectral analytic techniques
Source: PLoS One. 2022 Feb 25;17(2):e0264415. doi: 10.1371/journal.pone.0264415 (PMC8880644; doi:10.1371/journal.pone.0264415)
Supplement: Attachment — Submitted filename: Response to Reviewers3.docx [file pone.0264415.s005.docx]

Reviewer #1:

1. Please specify type of post hoc tests and their statistics used in the results sections "EEG data for NFT" - does it confirm F test results? if so for which data points? Please provide statistics for full groups in Fig 1. and Fig2 and accordingly corrects figures.

Response: Thank you for the reviewer’s suggestion. In this study, two-factor mixed analysis of variance (ANOVA) followed by Bonferroni post hoc testing was conducted on the mean relative alpha amplitude and amplitudes of the delta (1-3 Hz), theta (4-7 Hz), alpha (8-12 Hz), and beta (13-30 Hz) bands. The statistics in the "EEG data for NFT" are the results of the two-factor ANOVA followed by Bonferroni post hoc testing. We have modified the description of the type of post hoc test and statistic in the “Statistical analyses” part of the “Materials and methods” section.

(lines 244-247) *“**To evaluate the alpha NFT course and spectra, two-factor mixed analysis of variance (ANOVA) followed by Bonferroni post hoc testing was conducted on the mean relative alpha amplitude and amplitudes of the delta (1-3 Hz), theta (4-7 Hz), alpha (8-12 Hz), and beta (13-30 Hz) bands.”*

2. Also please also cite the literature behind your statements "The benefit of the bipolar recording was to reduce the possible artifacts of motion or eye blinks" - I did not find any example. Instead bipolar setting is used to monitor eye blinks - please check: <https://doi.org/10.1007/s10548-019-00707-x>

Response: Thank you for the reviewer’s suggestion. The bipolar recordings are used to monitor focal brain activity (Schomer D. L. & Da Silva, F. L. 2012). Bipolar recordings by placing electrodes around the eyes is used to measure the electrooculogram (EOG), that is the electrical potential caused by blinks and eye movements. Therefore, in this study, we used bipolar recording to monitor the focal brain activity around the central region. The bipolar recording can reduce any external artifact, such as movement artifacts or eye blinks (Marzbani et al., 2016, page 145, the bottom left side). We have added the citation behind the statement (line 132).

References

1. Schomer DL, Da Silva FL. Niedermeyer's electroencephalography: basic principles, clinical applications, and related fields. Lippincott Williams & Wilkins. 2012.
2. Marzbani H, Marateb HR, Mansourian M. Neurofeedback: a comprehensive review on system design, methodology and clinical applications. Basic Clin. Neurosci. 2016;7(2):143-58.
